# Gonorrhœa Easily Cured

**Published:** 1883-11-20

**Authors:** Z. T. Dellenbaugh

**Affiliations:** Cleveland, Ohio


### GONORRHŒA EASILY CURED.

BY Z. T. DELLENBAUGH, M. D., OF CLEVELAND, OHIO.

Founding an opinion on the recent text-books and treatises on this disease, one would imagine there had been little, if any, progress in its treatment. The young practitioner, without practical experience, who undertakes the management of gonorrhœal cases by the plan of treatment generally recommended in these works, with nauseating mixtures and conglomerate injections, will certainly be discouraged, and find his cases dragging along, or quit him, to become rounders. In cases of acute gonorrhœa I have, for eight or ten years, used carbonate of lithia to alkalinize the urine, and find the five-grain compressed tablets, one taken three times daily, very convenient, fulfilling every indication better than any other salt. I now rarely find it necessary to give any other remedy internally.

Should the case fail to respond to the following injection, and show marked improvement in two or three days, two sandal-wood oil capsules may be given, three times daily, for three or four days. The injection I have used in cases of acute and sub-acute gonorrhœa for more than a year, with the most gratifying results, especially to the patients, who have recovered in from two to seven days, and paid me from one to three visits, is the following—

℞ Resorcin.................................ʒ j,

Acid. boracic.............................gr. xx,

*Zinci acetatis*...........................gr. ¼–½,

Aquæ distillat..........................f. ℥ iv.

M. Of this solution two teaspoonfuls are injected three times daily. The germicides, resorcine and boracic acid are so slightly astringent, that it requires the additional zinc salt to restore capillary tonicity. This injection is quite or nearly painless.

In the treatment of the later stage of sub-acute and chronic gonorrhœa, without stricture or granuloma as a complicating factor, I have had the happiest results follow the use of the following injection—

R. Hydrargyri chloridi corrosivi...............gr. ¼ ss.
Zinci chloridi................................gr. ss–j,
Aquæ distillat..............................f. ℥ viij.

M. Sig. A tablespoonful to be injected well down into the urethra, three times daily.

Corrosive sublimate injections are by no means a recent addition to the list. The rationale of their use, however, is recent. As in the injection for acute cases, the germicidal constituent must be so sparingly used (otherwise it produces great pain and reactive inflammation), that I find it very advisable to combine a more astringent salt; and the chloride of zinc is the one I have selected, for obvious reasons. Without doubt, a mild injection of corrosive sublimate and chloride of zinc is destined to be the injection for sub-acute and chronic gonorrhœa.—*Clin. Record.*

